# Understanding the unbalance of interest in taxi market based on drivers' service profit margins

**DOI:** 10.1371/journal.pone.0198491

**Published:** 2018-06-18

**Authors:** Beibei Hu, Yawen Kong, Mengge Sun, Xianlei Dong, Gang Zong

**Affiliations:** 1 School of Management Science and Engineering, Shandong Normal University, Jinan, China; 2 School of Economics and Management, Beijing University of Technology, Beijing, China; South China University of Technology, CHINA

## Abstract

Nowadays, ride-hailing services have been established as a part of the urban transportation. Their arrival has remade the profit structure and resulted in the unbalance of interest in taxi market. Here, we establish the service models of taxis, carpooling, and car-hailing under “Internet +” from the perspective of profit margins, to perform a comparative analysis among the different services. Results: First, Profit margins are generally higher for short trips than for long trips, though empty cruise fee to a certain degree make up for the driver’s decreased profit margins. Second, the profit margin for carpooling is roughly 1.85 times that of ride-hailing, and 1.75 times that of taxis. This shows that the sharing economy has a certain advantage. Third, Profit margins are higher and fluctuations are lower on non-work days than on work days. At last, Profit margins are roughly 1.3 times higher on non-congested roads than on congested roads. The reduced profitability on congested roads makes it even harder to catch a ride during rush hours and on congested roads. We suggest that the relevant departments make appropriate efforts to make it more attractive for drivers to take on passengers during rush hours and on congested roads, and promote the sharing in the taxi market.

## Introduction

China’s taxi industry began to blossom in the early 1980s. Flexible, comfortable, always available, and carrying you right to your doorstep, taxis quickly became an indispensable service on China’s roads. As society and technology have become more advanced, the taxi industry is facing a new and rapid revolution reality. Didi Chuxing officially came into the market in China on June 1st, 2015. In just three months, this C2C (Customer to Customer) service accumulated 5.5 million carpool drivers and 8.4 million passengers, and saw a peak of 2.23 million daily orders. According to Forward Business and Intelligence of China (2016) [1], by October the same year, 53.7% of all rides were taken by people who had ordered them online. In July, 2016, the Ministry of Transport issued the *Temporary measures to manage online ride-hailing services* and *Guiding comments regarding deepening reform and promoting healthy development in the taxi industry*. These documents represent an approval of online ride-hailing by Chinese government. As the Internet and ride-hailing apps have become more prevalent, Didi Chuxing, Uber and other apps have entered the passenger transport market. Online platforms have added private cars on the supply side, satisfying consumer demands for high quality transportation services. As a new service mode in the “Internet + Transportation”, online ride-hailing has added new business opportunities and efficiently made use of idle vehicles. It has boosted the sharing economy and, to a certain degree, reduced the burden on China’s traffic and transportation infrastructure, making it easier for people in urban areas to catch a ride.

Having created a new reality in the taxi industry, these services present the traditional taxi industry with huge opportunities and challenges in terms of development and management. On the one hand, they have boosted information sharing in the taxi market and, to a certain degree, solved the information asymmetry problem. This increase in resource sharing and improved balance between supply and demand has improved the overall efficiency of the market. Roughly 97 million short rides are made in China every day, each costing an average of around 10 yuan. This put the market value at around 970 million yuan [2]. On the other hand, the new reality has caused a series of problems, such as vicious competition, price wars, and so on. The arrival of a large number of private cars has amounted to an attack on the traditional taxi market, and traditional taxis and ride-hailing cars have increasingly conflicting interests. This has led to a shrinking of the traditional taxi market, a fall in taxi drivers’ income, and many incidents of violence as a result of dissatisfaction. All of this has had an impact on the industry. To take one example, in 2015, Beijing’s taxis completed 588 million trips, 81 million fewer than the year before. According to the data from Beijing Traffic Development Research Center (2016) [3], the growth rate and number of trips were both the lowest in 10 years.

The competition and innovation in the taxi market are driven by a redistribution of the bulk of the profits in the market. Thus, it is necessary to have an accurate understanding on the market’s profit distribution structure in order to achieve efficient leadership and supervision. Traditional taxis and ride-hailing services are the main bodies in the market. The profits of the two are calculated in very different ways. In traditional taxi model [4], the driver’s profit mainly depends on how far and how long they drive. With the arrival of ride-hailing services, the traditional taxi model is no longer suitable for calculating profit margins. Existing models will have difficulties accurately balancing the differences in drivers’ profits among taxis, online carpooling services (hereafter referred to carpooling), and online ride-hailing services (hereafter referred to ride-hailing). In order to get an accurate measure of the situation in the taxi market and understand the profit margins distribution structures in it, it is important to create models of each “Internet +” taxi service model and calculate their profit margins.

This study is organized as follows: in section 2, we review relevant literature consisting of taxi service models and their profits. In section 3, we establish a basic service model, taxi service model, ride-hailing model, and carpooling model. In section 4, we provide model hypotheses and analyze differences in profit margins among different service mode, as well as the influence of work day/non-work day, congestion and other factors on profits. In the last section, we provide discussions and conclusions together with implications for further research.

## Literature review

The taxi market in China has always been plagued with an imbalance between supply and demand and information asymmetry. The demand for taxis has risen sharply, making it hard to catch a ride, while the ratio of empty cruise time has remained unchanged, meaning that drivers have trouble finding passengers. Statistics in 2015 show that 88.32% of all passengers wait for more than 10 minutes before catching a ride, while 53.77% of passengers have to wait more than 30 minutes [5]. This information disparity between passengers and drivers has made it hard to catch a ride, and caused a high rate of empty cruise time. Especially in the rush hour periods before and after work hours, as well as on congested roads, slow speeds cause a further decrease in supply. Drivers refusing to take on passengers or taking breaks will affect the relationship between supply and demand in the market, as well as their income [6–8]. A city’s transportation network is a massive and complex network containing complex, nonlinear relationships. Some scholars study taxi service modes from the system level [4]. For instance, by building a taxi service model from the network level, and describing how the taxi searches for passengers, it has been discovered that the taxi’s accessibility and capacity utilization rate will directly affect the supply and demand of taxis. When taxis spend a greater portion of their time carrying passengers, the passengers’ average waiting time increases [4]. The balance between supply and demand in the taxi market can be studied by investigating social surplus, company profits, and market structures [9–10]. Many papers have taken into consideration the important effect that congestion has on the taxi market to study the effect that distance, delays, and other factors have on incomes [11–12] and explored reform policies in the taxi market.

As positioning technology, information science, and mobile client services have become more advanced, people have begun to process data from GPS, GSM, and other sources relating to urban traffic [13]. For instance, empty cruise time has been used to evaluate taxi operation efficiency [14]; data on vehicles and traffic speed have been used to evaluate traffic conditions, and to survey intersections to find out how long vehicles take to pass through [15–17]; plans for passenger pick-ups have been optimized in order to improve drivers’ incomes [18–19]; passenger numbers and drivers’ search behavior have been evaluated [20–22]; the spatio-temporal dynamics of passenger activity have been studied [23]. Factors influencing drivers’ incomes include travel duration and distance [24–25], searching passengers and charges [24], the probability of finding passengers [26–27], area of work [28–29], etc. Although there already exist a large body of researches pertinent to the taxi market, the imbalance of supply and demand between drivers and passengers remains unresolved, and the taxi market remains relatively inefficient [30]. Many scholars have contributed by developing taxi recommendation systems [31–32], dispatch algorithms [33–34], and travel distribution plans [35–37]. This has helped optimize how information is shared to passengers and drivers and improved the balance between supply and demand in the market.

“Internet +” taxi service modes comprise a heavy blow to the traditional taxi market [38–40]. Market demand can no longer be met simply by cruising the streets. Current research on taxi service modes mainly focuses on business operations [41], supervision and regulation measures [42], the impact of ride-hailing software on taxi decision-making [43], and the danger posed to traditional taxi management systems by “Internet +” [44]. In the case of Didi Chuxing, people have analyzed the differences between the business modes of traditional taxis and ride-hailing services from the perspective of passengers [45]; created a platform for benign competition between ride-hailing services and taxis while studying the interest game and development trends of the taxi industry [46]. Scholars have also studied service quality, functions, and price on a micro level [47–48], payment methods and service evaluation [49], and resource distribution and service profits [50]. People analyze the key factors that influence the service quality and income disparity between traditional taxis and ride-hailing services, providing theoretical support for the regulation and management of urban taxi systems.

Previous literature has researched traditional taxi service models, service profit, and factors that influence drivers’ income. They have also analyzed taxi service modes and their business operations, ride-hailing services’ impact on the market, and other issues under “Internet +”. However, few papers have targeted the issue of how different taxi service modes make profits, and the traits of each profit mode. This paper sums up key factors that influence the profit of taxi services and compares the old and new service modes against each other. By targeting three different taxi services (taxis, carpooling, and ride-hailing), we establish the taxi service models under “Internet +” and explore the profit distributions of different service modes in the taxi market.

## Methodology

Currently, there are three main service models, taxis, ride-hailing, and carpooling in the taxi market. We can build the service models of taxis, ride-hailing and carpooling based on the basic service model for taxis [4]. The factors used to calculate cost and revenue for different service modes can be seen in Table 1.

**Table 1:** Factors used to calculate cost and revenue.

Service modes	Revenue	Cost
**Taxi**	Base rate, Mileage fee, Empty cruise fee, Low speed fee	Fuel cost, Monthly rents
**Ride-hailing**	Base rate, Mileage fee, Duration fee, Long distance fee, Low speed fee	Fuel cost, Information fee
**Carpooling**	Base rate, Mileage fee	Information fee, Fuel cost (for professional drivers)

### Basic model

The basic taxi service model takes the road network, the set of origins *I*, and the set of destinations *J* as its basis to study taxis’ service processes, and considers the entire trip from *i* ∈ *I* (the origins) to *j* ∈ *J* (the destinations). Given that *O* − *D* (origin-destination) of (*i*, *j*) ∈ (*I*, *J*), we set *F*_*ij*_ as the taxi fare from area *i* to *j*, then the taxi’s expected revenue when completing a trip *O* − *D* (*i* → *j*) can be shown in Eq (1):

$${\bar{F}}_{ij}=\frac{\sum_{\left(i,j\right)\in \left(I,J\right)}N_{ij}^{v}F_{ij}}{\sum_{\left(i,j\right)\in \left(I,J\right)}N_{ij}^{v}}$$ (1)

Here, $N_{ij}^{v}$ denotes the number of vehicles carrying passengers from *i* to *j* per unit time. The taxi’s expected time carrying passengers when completing one trip *O* − *D* (*i* → *j*) is:

$${\bar{h}}_{ij}=\frac{\sum_{\left(i,j\right)\in \left(I,J\right)}N_{ij}^{v}h_{ij}}{\sum_{\left(i,j\right)\in \left(I,J\right)}N_{ij}^{v}}$$ (2)

Here, *h*_*ij*_ denotes the time that the taxi travels from *i* to *j*. The taxi’s expected profit margin when completing a *O* − *D* (*i* → *j*) service can be shown as:

$${\bar{\pi }}_{ij}=\frac{{\bar{F}}_{i\to j}-(\phi_{0}{\bar{h}}_{ij}^{0}+\phi^{w}{\bar{w}}_{i}+\phi^{v}{\bar{h}}_{ij})}{{\bar{h}}_{ij}^{0}+{\bar{w}}_{i}+{\bar{h}}_{ij}}$$ (3)

In the above, ${\bar{h}}_{ij}^{0}$ denotes the average empty cruise time from other areas to *i*, ${\bar{w}}_{i}$ refers to the average waiting time at location *i*. *ϕ*_0_, *ϕ*^*w*^, *ϕ*^*v*^ represent fuel cost during empty cruise time per unit of time, waiting cost per unit of time, and fuel cost during the time spent carrying passengers per unit of time, respectively.

The fees considered in ride-hailing and carpooling are the same with those used in the traditional taxi service model (the basic model), and include base rate, mileage fee, empty cruise fee, low speed fee, and so on. Thus, we can build a model for ride-hailing, taxis, and carpooling under “Internet +” based on the basic model. In the following, we will provide three service models based on *O* − *D* (*i* → *j*), and then carry out a comparative analysis of the three different models’ profit margins.

### The taxi service model

Taxis calculate their fare as *F*_*ij*_ = *F*_*a*_ + *p*_*b*_(*S*_*ij*_ − *s*) + *F*_*low-speed*_ + *F*_*empty*_, where *F*_*a*_ is the base rate, *p*_*b*_ is the basic unit mileage price (price per kilometer exceeding the basic distance, usually begins pricing after 3 km for taxi), and *S*_*ij*_, *F*_*low-speed*_, *F*_*empty*_ refer to the distance, the low speed fee, and the empty cruise fee (i.e. long distance fee) accumulated during one service (*i* → *j*). *s* is the distance included in the base rate. Therefore, the expected revenue when completing one *O* − *D* (*i* → *j*) service is:

$$\begin{array}{l} {\bar{F}}_{ij}=\frac{\sum_{\left(i,j\right)\in \left(I,J\right)}N_{ij}^{v}F_{ij}}{\sum_{\left(i,j\right)\in \left(I,J\right)}N_{ij}^{v}}=\frac{\sum_{\left(i,j\right)\in \left(I,J\right)}N_{ij}^{v}[F_{a}+p_{b}(S_{ij}-s)+F_{low-speed}\text{+}F_{empty}]}{\sum_{\left(i,j\right)\in \left(I,J\right)}N_{ij}^{v}}\\ \;=F_{a}+{\bar{F}}_{low-speed}+{\bar{F}}_{empty}+p_{b}({\bar{S}}_{ij}-s) \end{array}$$ (4)

Where ${\bar{F}}_{low-speed}$,${\bar{F}}_{empty}$, ${\bar{S}}_{ij}$ refer to average low speed fee, average empty cruise fee, and average distance. ${\bar{F}}_{empty}$, ${\bar{S}}_{ij}$ are expressed as:

$$\begin{array}{l} {\bar{S}}_{ij}=\frac{\sum_{\left(i,j\right)\in \left(I,J\right)}N_{ij}^{v}S_{ij}}{\sum_{\left(i,j\right)\in \left(I,J\right)}N_{ij}^{v}},\\ {\bar{F}}_{empty}=p_{empty}\cdot ({\bar{S}}_{ij}-s\prime ) \end{array}$$ (5)

Here, *p*_*empty*_ and *s*′ represent the empty cruise fee per unit and the distance after which an empty cruise fee is charged.

If we disregard the difference in fuel cost between empty cruising and traveling with passengers, the expected profit margin when completing one trip could be:

$$\begin{array}{l} {\bar{\pi }}_{ij}=\frac{F_{a}+{\bar{F}}_{low-speed}+{\bar{F}}_{empty}+p_{b}({\bar{S}}_{ij}-s)-(\phi_{0}{\bar{h}}_{ij}^{0}+\phi^{w}{\bar{w}}_{i}+\phi^{v}{\bar{h}}_{ij})}{{\bar{h}}_{ij}^{0}+{\bar{w}}_{i}+{\bar{h}}_{ij}}-\phi_{M}\\ \;=\frac{F_{a}+\frac{p_{low-speed}\cdot {\bar{S}}_{ij}\cdot \psi \left(12\right)}{{\bar{V}}_{ij}}+p_{empty}({\bar{S}}_{ij}-s')+p_{b}({\bar{S}}_{ij}-s)-(\phi_{0}{\bar{h}}_{ij}^{0}+\phi^{w}{\bar{w}}_{i}+\phi^{v}{\bar{h}}_{ij})}{{\bar{h}}_{ij}^{0}+{\bar{w}}_{i}+{\bar{h}}_{ij}}-\phi_{M}\\ \;=(\frac{F_{a}-p_{b}\cdot s-p_{empty}\cdot s'}{{\bar{S}}_{ij}}+p_{b}+p_{empty})\bar{V_{ij}}\cdot \varepsilon_{0}+p_{low-speed}\cdot \varepsilon_{0}\cdot \psi \left(12\right)-\phi^{w}\varepsilon_{1}-\phi_{0}(1-\varepsilon_{1})-\phi_{M} \end{array}$$ (6)

Here, *ϕ*_0_, *ϕ*_*M*_, ${\bar{V}}_{ij}$ *ε*_0_, *ε*_1_ refer to fuel cost per unit time, monthly rents per unit time, average speed, capacity utilization rate (proportion of the time spent carrying passengers), and the proportion of the time the taxi spends waiting, respectively. Of these,

$$\phi_{\text{0}}=\frac{L\cdot S_{ij}\cdot p_{oil}}{100{\bar{h}}_{ij}},\varepsilon_{0}=\frac{{\bar{h}}_{ij}^{}}{{\bar{h}}_{ij}^{0}+{\bar{w}}_{i}+{\bar{h}}_{ij}},\varepsilon_{1}=\frac{{\bar{w}}_{i}}{{\bar{h}}_{ij}^{0}+{\bar{w}}_{i}+{\bar{h}}_{ij}}$$ (7)

Here, *L* and *p*_*oil*_ represent the fuel usage per 100 km and the fuel price, respectively. $\frac{L\cdot S_{ij}}{100}$ denotes the fuel usage from *i* to *j*. So $\frac{L\cdot S_{ij}\cdot p_{oil}}{100}$ refers to the fuel cost when completing an *O* − *D* (*i* → *j*) trip. Thus, the taxi’s expected profit margin when completing a trip is:

$${\bar{\pi }}_{ij}=\{\begin{array}{cc} \frac{F_{a}}{{\bar{S}}_{ij}}\bar{V_{ij}}\cdot \varepsilon_{0}+p_{low-speed}\cdot \varepsilon_{0}\cdot \psi \left(12\right)-\phi^{w}\varepsilon_{1}-\phi_{0}\left(1-\varepsilon_{1}\right)-\phi_{M} & S_{ij}\leq s\\ (\frac{F_{a}-p_{b}\cdot s}{{\bar{S}}_{ij}}+p_{b})\bar{V_{ij}}\cdot \varepsilon_{0}+p_{low-speed}\cdot \varepsilon_{0}\cdot \psi \left(12\right)-\phi^{w}\varepsilon_{1}-\phi_{0}\left(1-\varepsilon_{1}\right)-\phi_{M} & s<S_{ij}\leq s\prime \\ (\frac{F_{a}-p_{b}\cdot s-p_{empty}\cdot s\prime }{{\bar{S}}_{ij}}+p_{b}+p_{empty})\bar{V_{ij}}\cdot \varepsilon_{0}+p_{low-speed}\cdot \varepsilon_{0}\cdot \psi \left(12\right)-\phi^{w}\varepsilon_{1}-\phi_{0}\left(1-\varepsilon_{1}\right)-\phi_{M} & S_{ij}>s\prime \end{array}$$ (8)

### The ride-hailing service model

The ride-hailing service model calculates a driver’s revenue as: *F*_*ij*_ = *p*_*b*_*S*_*ij*_ + *p*_*c*_*h*_*ij*_ + *p*_*empty*_ (*S*_*ij*_ − *s*′), where *p*_*b*_, *p*_*c*_ represent the basic fare per unit of distance and the service fee (i.e. duration fee) per unit of time. Ride-hailing’s expected revenue for *O* − *D* (*i* → *j*) is:

$$\begin{array}{l} {\bar{F}}_{ij}=\frac{\sum_{\left(i,j\right)\in \left(I,J\right)}N_{ij}^{v}[p_{b}S_{ij}+p_{c}h_{ij}+p_{empty}\left(S_{ij}-s'\right)]}{\sum_{\left(i,j\right)\in \left(I,J\right)}N_{ij}^{v}}\\ \;=p_{b}{\bar{S}}_{ij}\text{+}p_{c}{\bar{h}}_{ij}+p_{empty}\left({\bar{S}}_{ij}-s'\right) \end{array}$$ (9)

The expected profit margin when completing a service is:

$$\begin{array}{l} {\bar{\pi }}_{ij}=\frac{{\bar{F}}_{ij}-F_{M}-(\phi_{0}{\bar{h}}_{ij}^{0}+\phi^{w}{\bar{w}}_{i}+\phi^{v}{\bar{h}}_{ij})}{{\bar{h}}_{ij}^{0}+{\bar{w}}_{i}+{\bar{h}}_{ij}}\\ \;=(p_{b}+p_{empty}-\frac{p_{b}\cdot s'+F_{M}}{{\bar{S}}_{ij}})\bar{V_{ij}}\cdot \varepsilon_{0}+p_{c}\cdot \varepsilon_{0}-\phi^{w}\varepsilon_{1}-\phi_{0}(1-\varepsilon_{1}) \end{array}$$ (10)

Here, *F*_*M*_ represents the information fee collected for every transaction by the online ride-hailing platform. If we disregard the difference in fuel cost between empty cruising and traveling with passengers, the expected profit margin when completing a trip is:

$${\bar{\pi }}_{ij}=\{\begin{array}{cc} \frac{F_{\text{min}}-F_{M}}{{\bar{S}}_{ij}}\bar{V_{ij}}\cdot \varepsilon_{0}-\phi^{w}\varepsilon_{1}-\phi_{0}(1-\varepsilon_{1}) & F_{ij}\leq F_{\text{min}}\\ \left(p_{b}-\frac{F_{M}}{{\bar{S}}_{ij}})\cdot \bar{V_{ij}}\cdot \varepsilon_{0}+p_{c}\cdot \varepsilon_{0}-\phi^{w}\varepsilon_{1}-\phi_{0}(1-\varepsilon_{1}\right) & F_{ij}>F_{\text{min}},0<S_{ij}\leq s\prime \\ \left(p_{b}+p_{empty}-\frac{p_{empty}\cdot s\prime +F_{M}}{{\bar{S}}_{ij}})\cdot \bar{V_{ij}}\cdot \varepsilon_{0}+p_{c}\cdot \varepsilon_{0}-\phi^{w}\varepsilon_{1}-\phi_{0}(1-\varepsilon_{1}\right) & F_{ij}>F_{\text{min}},S_{ij}>s\prime \end{array}$$ (11)

Here, *F*_min_ represents the lowest revenue for each ride.

### The carpooling service model

For carpooling, there are two types of fares: normal fare and shared ride fare. The normal fare is calculated: *F*_*ij*_ = *F*_*a*_ + *p*_*b*_ (*S*_*ij*_ − *s*). Here, *F*_*a*_, *p*_*b*_ represent the basic fare and the price of per unit distance (price per kilometer exceeding the basic distance, usually begins pricing after 2 km for carpooling). Thus, the expected revenue for an *O* − *D* (*i* → *j*) trip is:

$${\bar{F}}_{ij}=\frac{\sum_{\left(i,j\right)\in \left(I,J\right)}N_{ij}^{v}[F_{a}+p_{b}(S_{ij}-s)]}{\sum_{\left(i,j\right)\in \left(I,J\right)}N_{ij}^{v}}=F_{a}+p_{b}\cdot ({\bar{S}}_{ij}-s)$$ (12)

In this case, the expected profit margin when completing a trip is:

$$\begin{array}{l} {\bar{\pi }}_{ij}=\frac{{\bar{F}}_{ij}-(\phi_{0}{\bar{h}}_{ij}^{0}+\phi^{w}{\bar{w}}_{i}+\phi^{v}{\bar{h}}_{ij})}{{\bar{h}}_{ij}^{0}+{\bar{w}}_{i}+{\bar{h}}_{ij}}\\ \;=(\frac{F_{a}-p_{b}\cdot s}{{\bar{S}}_{ij}}+p_{b})\bar{V_{ij}}\cdot \varepsilon_{0}(1-P_{F_{M}})-\phi^{w}\varepsilon_{1}-\phi_{0}\left(1-\varepsilon_{1}\right) \end{array}$$ (13)

Here, $P_{F_{M}}$ represents the percentage of the information fee charged by online ride-hailing platform. There are part-time carpooling drivers as well as full-time ones. Different from full-time drivers, part-time carpooling drivers pick up passengers along a certain route, whether or not they actually pick up passengers will not affect their fuel cost. Thus, in this case let *ϕ*_0_ = 0, otherwise *ϕ*_0_ ≠0. In general, carpooling drivers will take orders in advance and arrive early to pick up passenger(s). As a result, there is cost related to waiting time *ϕ*_*w*_. A carpooling driver’s expected profit margin when completing a trip can be as shown in Eq (14), where *k* denotes the proportion of full-time carpooling drivers.

$${\bar{\pi }}_{ij}=\{\begin{array}{ll} \frac{F_{a}}{{\bar{S}}_{ij}}\bar{V_{ij}}\cdot \varepsilon_{0}(1-P_{F_{M}})-k\phi_{0}(1-\varepsilon_{1}\text{)}-\phi^{w}\varepsilon_{1} & \begin{array}{r} 0<S_{ij}\leq s \end{array}\\ (\frac{F_{a}-p_{b}\cdot s}{{\bar{S}}_{ij}}+p_{b})\bar{V_{ij}}\cdot \varepsilon_{0}(1-P_{F_{M}})-k\phi_{0}\text{(1-}\varepsilon_{1}\text{)}-\phi^{w}\varepsilon_{1} & \begin{array}{r} S_{ij}>s \end{array} \end{array}$$ (14)

For the other service mode, the shared fares are calculated for all of the separate stretches that the passengers travel and added together. Thus, when completing one *O* − *D* (*i* → *j*) trip, let the number of individual orders be *λ*, then a carpool driver’s expected revenue for an *O* − *D* (*i* → *j*) service is:

$$\begin{array}{l} {\bar{F}}_{ij}=\lambda \frac{\sum_{\left(i,j\right)\in \left(I,J\right)}N_{ij}^{v}[F_{a}+p_{b}\cdot (s_{1}-s)+p_{b}^{1}\cdot (S_{ij}-s_{1})]}{\sum_{\left(i,j\right)\in \left(I,J\right)}N_{ij}^{v}}\\ \;=\lambda [F_{a}+p_{b}\cdot (s_{1}-s)+p_{b}^{1}\cdot ({\bar{S}}_{ij}-s_{1})] \end{array}$$ (15)

Here, *p*_*b*_ and $p_{b}^{1}$ represent a unit price for trips above *s* and *s*_1_ km, respectively (i.e. mileage fee).*s* and *s*_1_ are constants, where *s* being the distance included in the base rate, and *s*_1_ being 10 km. In this case, the expected profit margin when completing a trip is:

$$\begin{array}{l} {\bar{\pi }}_{ij}=\frac{\left(1-P_{F_{M}}){\bar{F}}_{ij}-(k\phi_{0}{\bar{h}}_{ij}^{0}+\phi^{w}{\bar{w}}_{i}+k\phi^{v}{\bar{h}}_{ij}\right)}{{\bar{h}}_{ij}^{0}+{\bar{w}}_{i}+{\bar{h}}_{ij}}\\ \;=\lambda (\frac{F_{a}+p_{b}(s_{1}-s)-p_{b}^{1}\cdot s_{1}}{{\bar{S}}_{ij}}+p_{b}^{1})\bar{V_{ij}}\cdot \varepsilon_{0}(1-P_{F_{M}})-k\phi_{0}\text{(1-}\varepsilon_{1}\text{)}-\phi^{w}\varepsilon_{1} \end{array}$$ (16)

For different trips, the expected profit margin can be described in Eq (17):

$${\bar{\pi }}_{ij}=\{\begin{array}{ll} \lambda \frac{F_{a}}{{\bar{S}}_{ij}}\bar{V_{ij}}\cdot \varepsilon_{0}(1-P_{F_{M}})-k\phi_{0}\text{(1-}\varepsilon_{1}\text{)}-\phi^{w}\varepsilon_{1} & \text{0}<S_{ij}\leq s\\ \lambda (\frac{F_{a}-p_{b}\cdot s}{{\bar{S}}_{ij}}+p_{b})\bar{V_{ij}}\cdot \varepsilon_{0}(1-P_{F_{M}})-k\phi_{0}\text{(1-}\varepsilon_{1}\text{)}-\phi^{w}\varepsilon_{1} & s<S_{ij}\leq s_{1}\\ \lambda (\frac{F_{a}+p_{b}(s_{1}-s)-p_{b}^{1}\cdot s_{1}}{{\bar{S}}_{ij}}+p_{b}^{1})\bar{V_{ij}}\cdot \varepsilon_{0}(1-P_{F_{M}})-k\phi_{0}\text{(1-}\varepsilon_{1}\text{)}-\phi^{w}\varepsilon_{1} & S_{ij}>s_{1} \end{array}$$ (17)

## Materials

### Data gathering

According to the statistical data issued by traffic police detachment of Jinan Public Security Bureau in June, 2017[51], the congested areas mainly consist of Lixia District, Shizhong District, Licheng District, Tianqiao District and Huaiyin District. Nineteen routes in these districts in Jinan city (as shown in Table 2) are sampled to collect related data. Mileage and travel time for each trip are retrieved using Baidu Map and AMAP. The data have been collected between April 2, 2017 and June 5, 2017, from 7 am to 10 pm, including 425 work days, 175 weekends, and 3 public holidays (Tomb Sweeping Day, Dragon Boat Festival, and May Day). In total, we gather 17,160 data points, and each data point represent the expected time that a vehicle spends passing through a designated stretch of road.

**Table 2:** Origins, destinations, and length of stretches of the 19 routes.

No.	Origin	Destination	Distance (km)
**1**	Spouting Spring Park- East Gate	Shandong News Mansion	1.8
**2**	Jinan Station	Stomatological Hospital of Shandong University	2.0
**3**	Shandong Sports Center	Jinan Cemetery of Revolutionary Martyrs	2.1
**4**	Spouting Spring Park- East Gate	Jinan Xuefu Hotel	2.2
**5**	Spouting Spring Park- East Gate	Shandong Sports Center	2.7
**6**	Xiuyuan Community	Harmony Mall	2.8
**7**	Shandong Sports Center	Jinan University—East Campus	3.6
**8**	Black Tiger Spring	Jinan University—East Campus	4.3
**9**	Xiuyuan Community	Jinan University—East Campus	4.3
**10**	Jinan Building	Qianfoshan Hospital	4.4
**11**	Jinan University—East Campus	Qianfoshan Hospital	4.4
**12**	Black Tiger Spring	Jinan Cemetery of Revolutionary Martyrs	5.4
**13**	Yuyuan Community	Shandong Sports Center	5.8
**14**	Erqixincun South Road North Exit	Black Tiger Spring	6.1
**15**	Jinan Station	Shandong Chest Hospital	6.2
**16**	Shandong Ophthalmic Hospital	Shandong Water ResourcesDepartment	6.3
**17**	Harmony Mall (Huaiyin Community onJingshiRoad)	Shandong Chest Hospital	8.6
**18**	Yuyuan Community	Shandong Chest Hospital	8.8
**19**	Xiuyuan Community—Bus Stop	Shandong Chest Hospital	11.1

Using the document *Notice Regarding Adjustments to the Pricing of Passenger Transport in Jinan* issued by Jinan Commodity Price Bureau in 2014 and the documents *User Manual* and *Driver Manual* released by Didi Chuxing, we have retrieved price calculation standards for different service models, as shown in Table 3:

**Table 3:** Charge standard for taxis and ride-hailing services in Jinan.

	Category		Fare		
**Taxi**	Base rate (0–3 km)		9 yuan		
	Mileage fee		1.5 yuan/km		
	Low speed fee		Below 12 km/h: added 0.2 yuan/minute (excl. empty cruise fee)		
	Empty cruise fee		After 6 km carrying passengers, 0.75 yuan/km		
**Carpooling**	Normal		Shared ride		
	Distance				
No. of passengers	0–2 km	Over 2 km	0–2 km	2–10 km	Over 10 km
1	5 yuan	1.2 yuan/km	4 yuan	0.96 yuan/km	0.72 yuan/km
2	5 yuan	1.2 yuan/km	4 yuan	0.96 yuan/km	0.96 yuan/km
3	6 yuan	1.44 yuan/km	5.5 yuan	1.32 yuan/km	1.32 yuan/km
4	7 yuan	1.68 yuan/km	7 yuan	1.68 yuan/km	1.68 yuan/km
**Ride-hailing**	Passenger’s fare		Driver’s charge		
Mileage fee	1.5 yuan/km		1.2 yuan/km		
Duration fee	0.33 yuan/minute		0.26 yuan/minute		
Empty cruise fee	0.5 yuan/km (over 10 km)		0.4 yuan/km (over 12 km)		
Minimum charge	8 yuan		4.8 yuan		

**Notes**: (1) According to the document *Notice Regarding Increased Vehicle Lease Fees for 2nd Generation Colored Taxis* released by Jinan Commodity Pricing Bureau, taxi drivers must pay their taxi company a monthly rent (management fee included) for roughly 4,050 yuan. (2) According to driver’s credit status, Didi Chuxing collects a 10%-20% information charges from carpool drivers. (3) Since most carpool drivers are in good financial standing, we take 10% as the charge rate of information fee. (4) Didi Chuxing collects a 0.5-yuan information charge from every trip made by a car-hailing driver.

### Model hypotheses

In order to simplify the calculations, the empirical study is made based on the following hypotheses:

1. We do not take into consideration the difference of capacity utilization rate between taxis and ride-hailing cars, but set the intervals of the capacity utilization rate during rush hours and normal hours for taxis and ride-hailing cars at [0.8, 1.0] and [0.7, 0.9], respectively. We use random sampling to determine the capacity utilization rate at different time of the day and on different routes based on the uniform distribution.
2. We do not take into consideration any difference in the average speed and the fuel cost of taxis and ride-hailing cars either they carry passengers or cruise empty.
3. We do not take into consideration the work done by any drivers at night.
4. We do not take into consideration the cost caused by waiting.
5. We assume that the speed of taxis and ride-hailing cars follow a normal distribution, and their service distance is estimated during low speed driving accordingly.
6. We assume an inverse linear relationship between the car’s speed and its fuel consumption. Specifically, we assume that during the service, fuel consumption at 12 km/h is double that at 60 km/h. In which, 12 km/h is the boundary for low speed service, while 60 km/h is the upper speed limit in urban areas in most cases.
7. We assume full-time carpooling drivers account for about 50 percent in the carpooling market. So let *k* = 50% in Eqs (14)–(17). Meanwhile, in the shared cases, we suppose a carpooling driver picks up 2 individual orders when the driver completes an *O* − *D* (*i* → *j*) trip, i.e. let *λ* = 2.

In the following, we will calculate the service profit margins of taxis and online ride-hailing cars.

### Calculation of service profit margins

#### Calculating average speed

**Fig 1:**
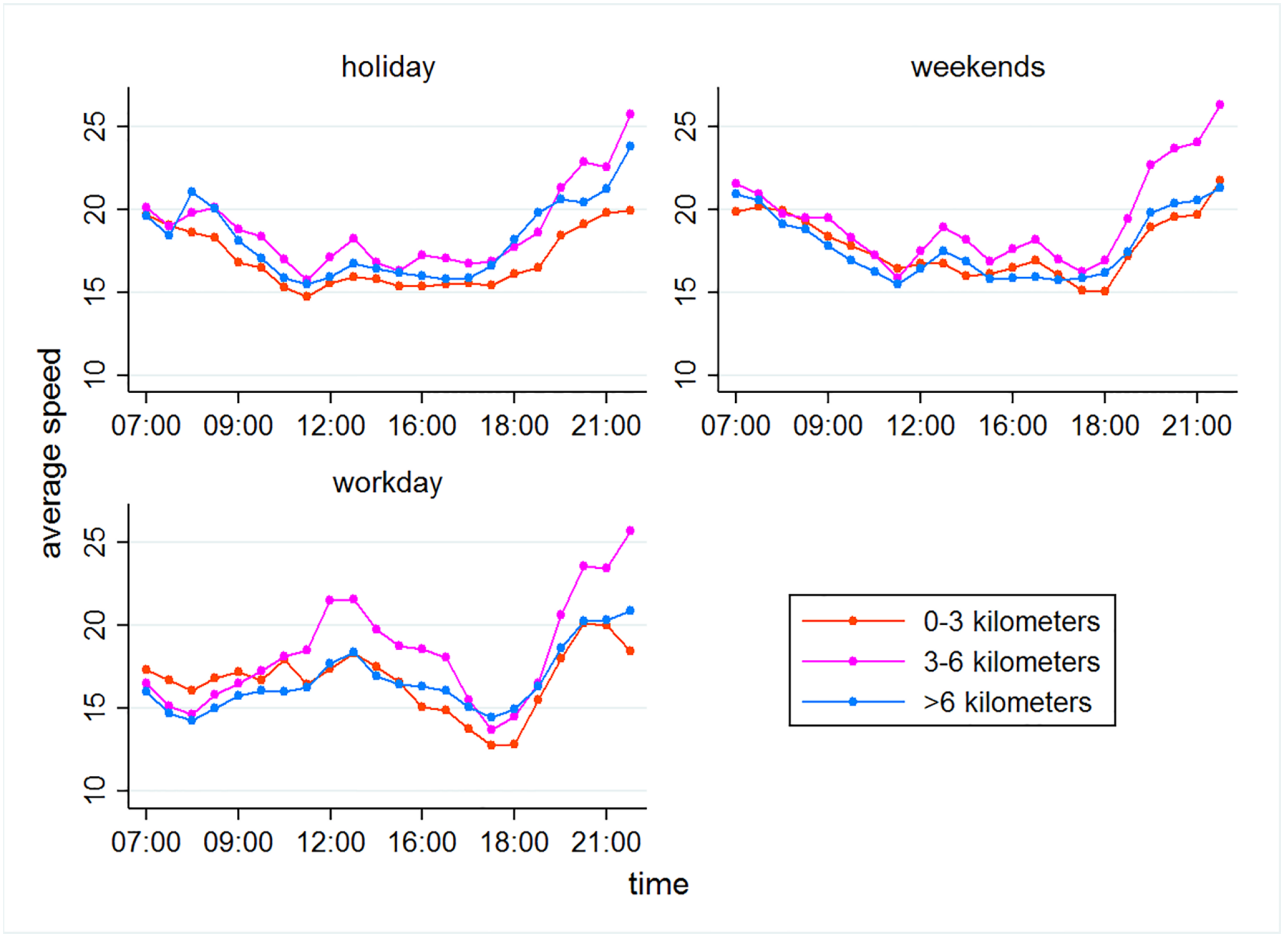
Daily changes in travel speeds.

The traffic capacity of each stretch of road could be represented by the average traffic speed at different time of the day. We use $D_{i}=\left(D_{i}^{w_{1}},D_{i}^{w_{2}},D_{i}^{h}\right)$ to represent the sample quantity from each stretch of road *i*, where $D_{i}^{w_{1}}$, $D_{i}^{w_{2}}$ and $D_{i}^{h}$ represent the sample quantity from the stretch of road *i* on work days, weekends, and public holidays. Let *S*_*i*_ = (*i* = 1, 2,⋯19) represents distance, and $T_{ij}^{\left(d\right)}$ (*j* = 7:00, 7:30, 8:00,⋯22:00; *d* = 1, 2,⋯*D*_i_) represents the time *j* when a vehicle (sample *d*) passes through the stretch of road *i*. A car’s instantaneous speed at time *j* when it passes through stretch *i* can be expressed as ${\bar{v}}_{ij}^{\left(d\right)}=\frac{s_{i}}{T_{ij}^{\left(d\right)}}$. Thus, by analyzing all the samples, a car’s average speed at time *j* when it passes through stretch *i* can be expressed as ${\bar{v}}_{ij}=\frac{\sum_{d=1}^{D_{i}}v_{ij}^{\left(d\right)}}{D_{i}}$. Given that the traffic situation is different during work days, weekends, and public holidays, we calculate the average speed for these days respectively, that is, ${\bar{v}}_{ij}^{w_{1}}=\frac{\sum_{d=1}^{D_{i}^{w_{1}}}v_{ij}^{\left(d\right)}}{D_{i}^{w_{1}}},{\bar{v}}_{ij}^{w_{2}}=\frac{\sum_{d=1}^{D_{i}^{w_{2}}}v_{ij}^{\left(d\right)}}{D_{i}^{w_{2}}},{\bar{v}}_{ij}^{h}=\frac{\sum_{d=1}^{D_{i}^{h}}v_{ij}^{\left(d\right)}}{D_{i}^{h}}$. We divide the stretches of these 19 routes into three categories according to how fares are calculated: Stretches of 0–3 km (6 stretches), 3–6 km (7 stretches), and over 6 km (6 stretches). Fig 1 shows how the average speed of vehicles changes over time (after separating them into categories).

Fig 1 tells us that the daily changes in average speed are roughly the same on work days, weekends and public holidays, creating a *W*-like shape. The drop of speed is more obvious during morning and evening rush hours. For the 19 routes, rush hours are from 7:30 to 8:30 and 17:00 to 18:30 on work days. However, during weekends and holidays, the morning rush hour occurs around 10:00 to 11:00, while the evening rush hour is mostly the same as that during work days.

#### Calculating low speed fees

Next, we will calculate the low speed (below 12 km/h) fees for cars on each stretch of road. Based on the assumption that the speed *V*_*ij*_ follows a normal distribution, that is, $V_{ij}\sim N\left({\bar{V}}_{ij},\sigma^{2}\right)$, ${\bar{V}}_{ij}=\frac{{\bar{S}}_{ij}}{{\bar{h}}_{ij}}$, let *ψ*(12) denotes a cumulative probability, in the normal distribution with an expectation of ${\bar{V}}_{ij}$ and a standard deviation of *σ* (i.e.*ψ*(12) = p(*v*≤12). Then the expected low speed fee can be expressed as:

$${\bar{F}}_{low-speed}=\frac{p_{low-speed}\cdot {\bar{S}}_{ij}\cdot \psi \left(12\right)}{{\bar{V}}_{ij}}$$ (18)

In which, *ψ*(12) can be obtained by using the Taylor series in Eq (19):

$$\psi (v)=1/2+\varphi (v)(v+v^{3}/3+v^{5}/(3\times 5)+v^{7}/(3\times 5\times 7)+\cdots )$$ (19)

Here, *φ*(*v*) represents the probability density function for the normal distribution.

We postulate that the capacity utilization rate for taxis and online ride-hailing cars during normal hours is [0.7, 0.9], and [0.8, 1.0] during rush hours. We choose capacity utilization rate along different stretches at different time based on uniform distribution. Then we can calculate the service profit margins of taxis, ride-hailing cars, and carpooling cars (normal ride or shared ride) using the Eqs (8), (11), (14) and (17).

## Results and discussion

### Comparative analysis of the profit margins based on trip length

**Fig 2:**
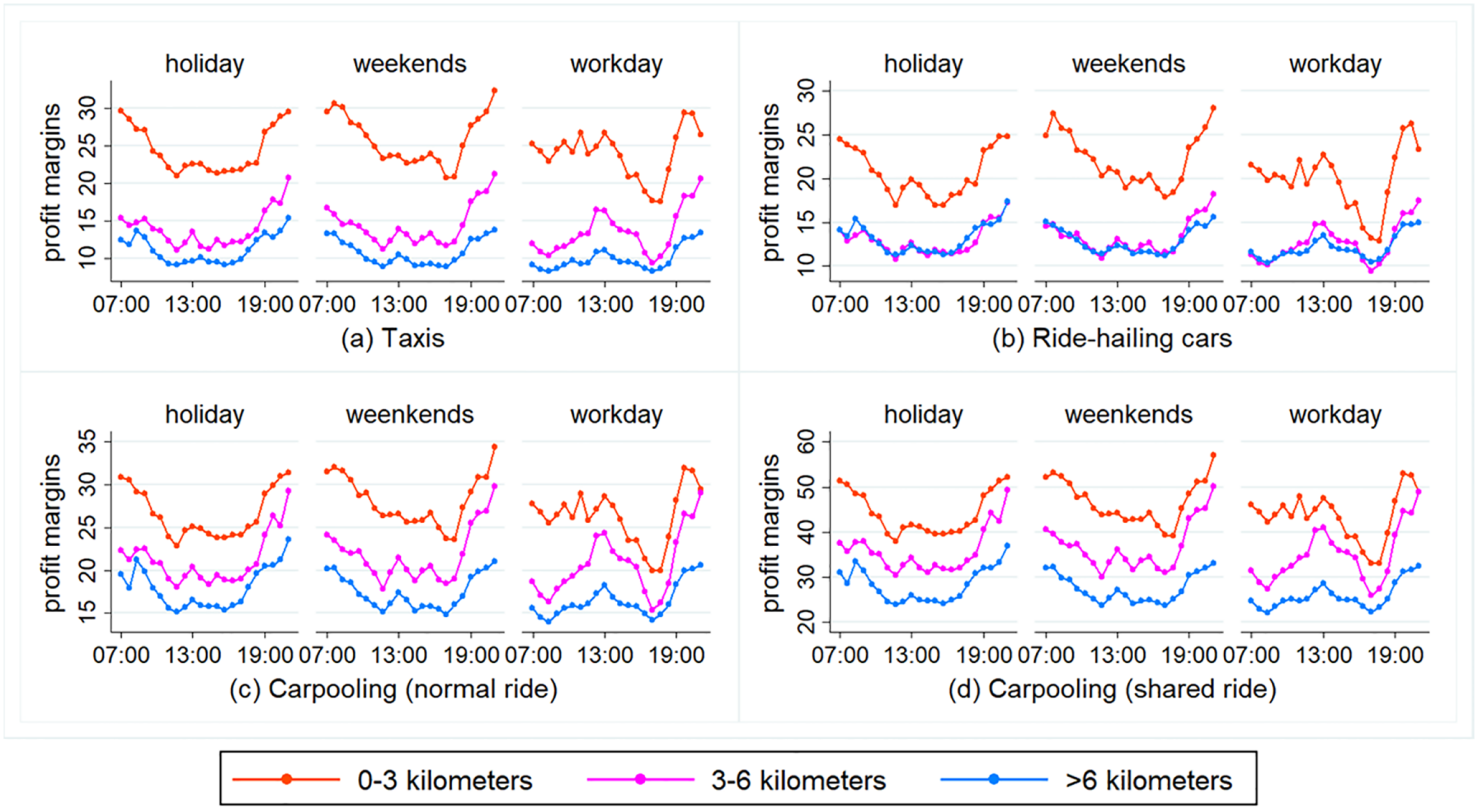
Comparing profit margins of taxis (online ride-hailing cars) on different trip lengths.

The profit margins of the taxis and online ride-hailing cars on different trip lengths (0–3 km, 3–6 km, over 6 km) are shown in Fig 2. Overall, short trips generate higher profit margins than long trips. The longer the trip, the more profit diminishes. During the weekends, for instance, the daily average profit margin of taxis for trips of 0–3 km, 3–6 km, and over 6 km are 25.87 yuan, 14.43 yuan, and 10.80 yuan per hour, respectively. Ranked from the highest profit margin to the lowest, the services are carpooling (multiple passengers and single passenger), taxis, and ride-hailing cars. For instance, during a work day, these services have an average profit margin per hour of 34.73 yuan, 21.13 yuan, 15.82 yuan, and 14.88 yuan respectively. Carpooling has the highest profit margin, and it becomes even higher when carrying multiple passengers.

### Comparing the profit margins during work days, weekends, and public holidays

**Fig 3:**
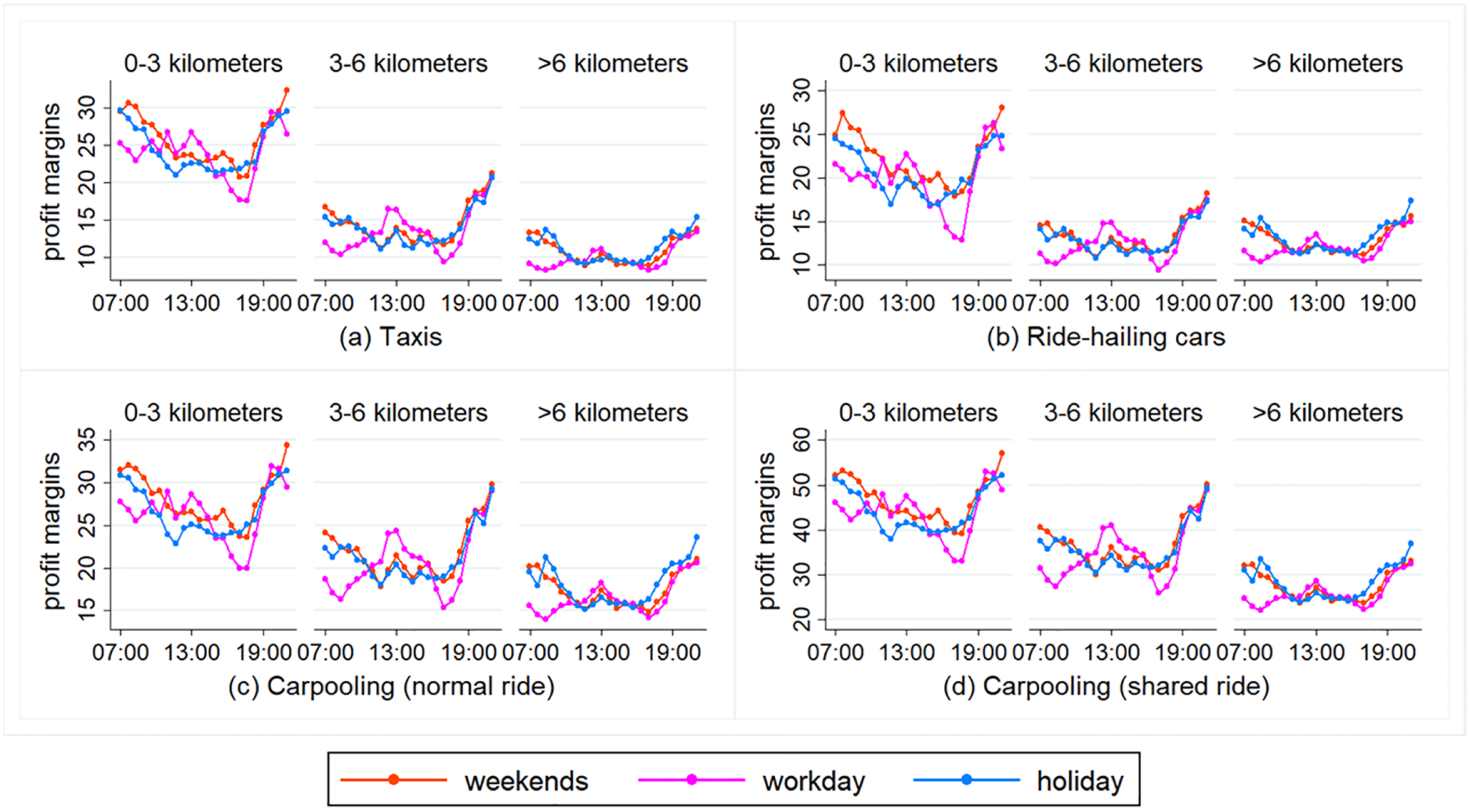
Comparing the profit margins during work days, weekends, and public holidays.

The profit margins of taxis and online ride-hailing cars are influenced by whether it is a work day or not, as shown in Fig 3. Overall, profit margins are greater during non-work days. However, as the distance of the trips increases, the difference in profit margins between work days and non-work days decreases. For instance, on trips of 0–3 km and 3–6 km, the profit margin for taxis is 1.88 yuan, which is 0.88 yuan higher on non-work days than on work days. Besides, taxis and ride-hailing cars see greater fluctuations in their profit margins on work days than on non-work days. The profit margins are distinctly lower during rush hours than during other periods of time. Normally, the lowest profit margins during the morning rush hour occur around 08:00, while the lowest profit margins during the evening rush hours appear from 17:30 to 18:00. To take 0–3 km trips on work days as an example, ride-hailing cars see their lowest profits for these trips during the morning rush hour at 08:00, at 19.74 yuan per hour. This is 0.18 yuan per hour below the daily average. The lowest profit margin during the evening rush hour appear at 18:00, at 12.84 yuan per hour, a whole 7.07 yuan per hour below the daily average.

### Effects of congestion on profit margins

**Fig 4:**
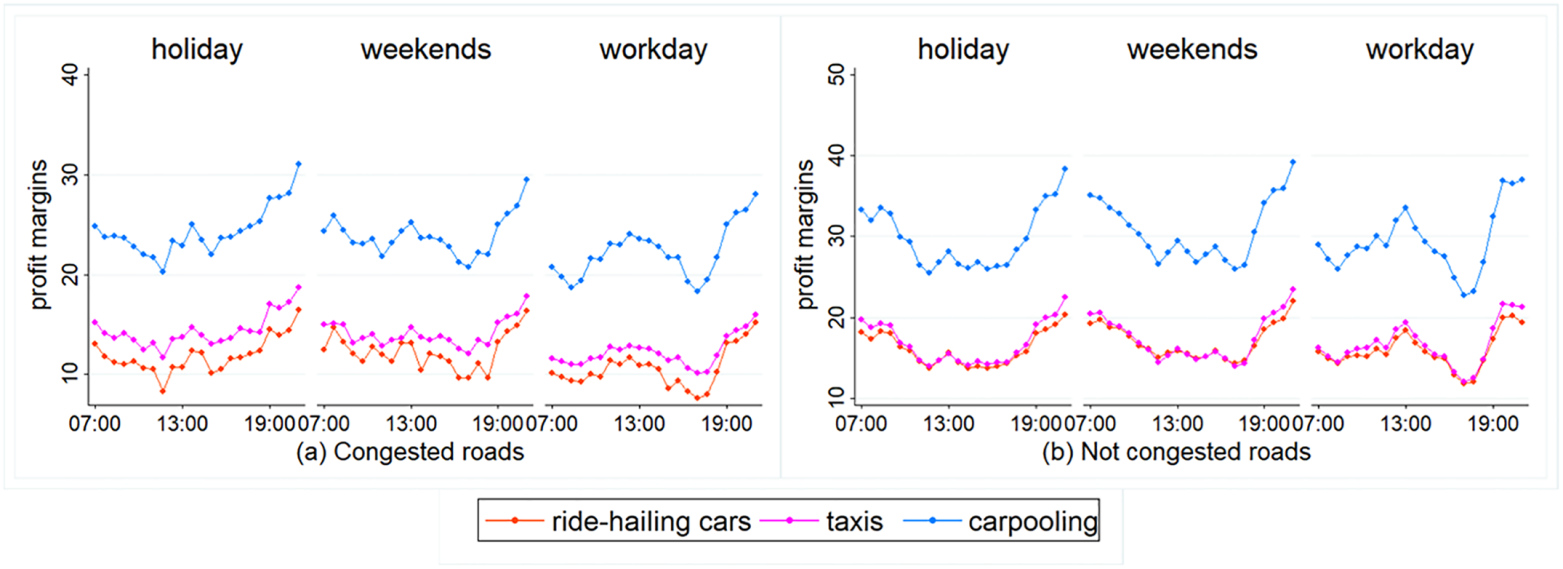
Comparing the effects of congestion of profit margins.

We define a stretch of road as congested if the daily average speed (from 7am to 10pm) there is below 15 km/h. If this is not the case, we do not define it as congested. Data show that 4 stretches of road are congested during work days, while 3 are congested during non-work days (the rest are not congested). Fig 4 shows the changes in profit margins on both congested and non-congested roads.

Fig 4 tells us that congested roads see smaller fluctuations in vehicle speed during the different time periods, and that profit margins are more stable. Conversely, non-congested roads see greater fluctuations in profit margins, especially between rush hours and normal hours. Overall, carpooling cars achieve the highest profit margins on congested roads, followed by taxis, while ride-hailing cars come last. On non-congested roads, taxis and ride-hailing cars achieve roughly the same profit margins. In general, the profit margins are markedly higher on non-congested roads than on congested roads. To take one example, on work days, carpooling cars, taxis, and ride-hailing cars achieve average daily profit margins of 22.23 yuan per hour, 12.21 yuan per hour, and 10.59 yuan per hour respectively on congested roads. On non-congested roads, these numbers are 29.44 yuan per hour, 16.63 yuan per hour, and 15.86 yuan per hour. On non-congested roads, the effect of rush hours is greater on work days. Profit margins fall markedly during rush hours. For instance, on work days, profit margins for carpooling cars, taxis, and ride-hailing cars fall 21.38%, 20.63%, and 17.70% respectively during rush hours, compared with normal hours.

We use Eq (20) to calculate the difference *D*_*π*_ in profit margins between congested and non-congested roads and further analyze the effect of congestion on the different services’ profit margins.

$$D_{\pi }=\pi_{not-congested}-\pi_{congested}$$ (20)

**Fig 5:**
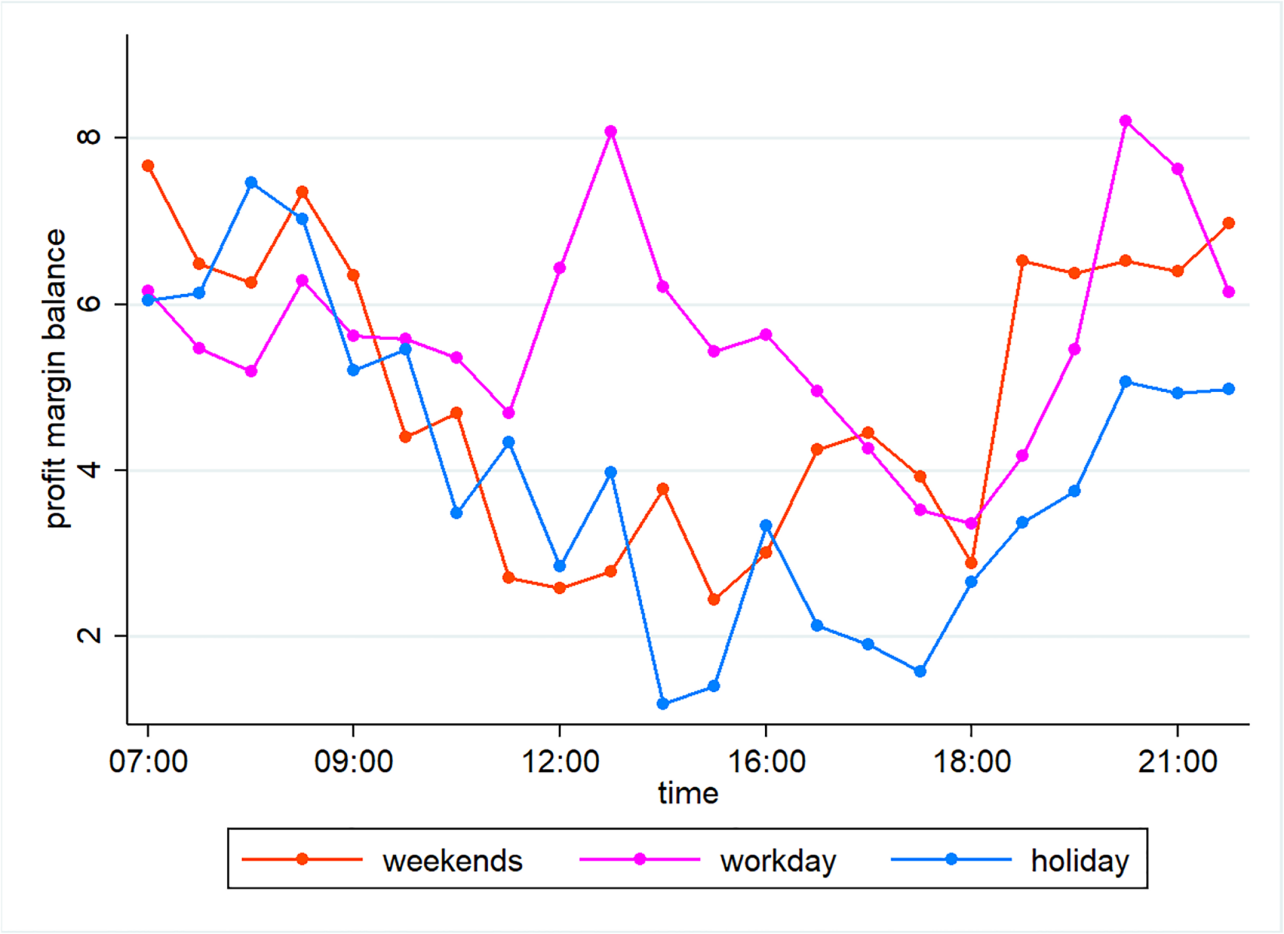
The changes in profit margins’ difference over time on congested and non-congested roads.

Here, *π*_*not-congested*_ and *π*_*congested*_ represent average profit margins on non-congested roads and congested roads. These are obtained by calculating the mean value of the profit margins for the different service modes on congested roads and non-congested roads. Fig 5 shows the differences in profit margins on congested and non-congested roads. From Fig 5, we can see that changes in profit margins are markedly different on work days and non-work days. On work days, the difference in profit margins on congested and non-congested roads is rather small during rush hours; however, during normal hours, non-congested roads see markedly higher profit margins, with the largest difference being 8.21 yuan per hour.

## Conclusions

In this paper, we establish service models for taxis, ride-hailing and carpooling under “Internet +”. We calculate the profit margins of the different models and conduct an empirical study on 19 routes in Jinan city in Shandong province, China. Our conclusions are as follows:

Overall, short trips generate higher profits than long trips. The longer the trip, the more profit diminishes. The empty cruise fee lowers the negative effect of a long ride on a driver’s profit. Ranking the services from the highest to the lowest profit margins, we get carpooling (multiple passengers), carpooling (single passenger), taxis and ride-hailing cars. Carpooling cars achieve higher profit margins, because carpool drivers can pick up shared ride orders as well as the fuel cost of part of part-time drivers is not calculated into the costs. This creates a high profit margin and tells us that the market for shared transportation has a bright outlook. Specifically, the profit margin of carpooling cars is roughly 1.85 times that of ride-hailing cars and 1.75 times that of taxis. By promoting sharing economy in the taxi and online ride-hailing market, such as encouraging ride sharing in taxis and carpooling cars, one can efficiently reduce congestion while improving incomes. It can also advance innovation and sustainable development in the taxi and ride-hailing markets.

Profit margins are usually lower on work days than on non-work days. In other words, drivers see higher profits during weekends and public holidays. Taxis (online ride-hailing cars) see greater fluctuations in their profit margins on work days than on non-work days, especially during rush hours. Speed has a clear impact on profit margins. Drivers that maintain high speeds while providing their service achieve higher profit margins. The data analysis shows that profit margins on non-congested roads are roughly 1.3 times than those on congested roads. Low profit margins on congested roads would cause some drivers refusing to pick up passengers along the roads. Combined with an already heightened demand for rides during rush hours, this makes it even harder for people to catch rides during these periods. Reasonable plans should be laid to increase the availability of taxis (online ride-hailing cars) along congested roads, especially during rush hours. An appropriate low speed fee should be set during rush hours for taxis (online ride-hailing cars), and a reasonable charging system should be set up for ride-hailing platforms. This would make it more attractive for drivers to pick up passengers during rush hours and along congested roads.

In this paper, we comparatively analyze the profit margins of different service modes to arrive at a series of valuable conclusions. The paper does, however, contain some shortcomings needing to be improved. First of all, due to limitations of data collection, we have not taken into consideration any differences in the cost of waiting among the different service modes. Furthermore, because the capacity utilization rate is obtained by sampling uniform distribution, our calculations may contain certain systematic deviations. Second, we use ideal normal distribution to fit the changes of speed to calculate low speed fees. We use a simplified and linear model to fit the relationship between fuel cost and vehicle speed to calculate the fuel costs at different speeds. The reality on the ground, however, is complex, and the traffic conditions are changeable. In our future research, we will improve our data collection methods as well as accurately quantify the capacity utilization rate, driver waiting cost, fuel cost and so on, making our research even more objective and accurate.

## Supporting information

S1 File Holiday data. (CSV)

S2 File Weekends data. (CSV)

S3 File Workdays data. (CSV)
